# Genome-wide association study reveals novel QTLs and candidate genes for panicle number in rice

**DOI:** 10.3389/fgene.2024.1470294

**Published:** 2024-11-05

**Authors:** Jiangfan Guo, Wenbin Wang, Wei Li

**Affiliations:** ^1^ School of Life Sciences, Shaanxi Normal University, Xi’an, China; ^2^ Research Institute of Life Sciences Computing, Zhejiang Lab, Hangzhou, China

**Keywords:** panicle number, rice, genome-wide association study, quantitative trait loci, candidate genes

## Abstract

**Introduction:**

Panicle number (PN) is one of the three key yield components in rice, maintaining stable tiller and PN is a crucial characteristic of an ideal plant architecture. Understanding the molecular mechanisms underlying PN is essential for breeders aiming to improve rice yield.

**Methods:**

To dissect the genetic architecture of panicle number, a genome-wide association study (GWAS) was conducted in 411 *japonica* rice varieties. GWAS analysis was carried out with the mixed linear model using 743,678 high-quality SNPs.

**Results:**

Over two experimental years, we detected a total of seven quantitative trait loci (QTLs), located on chromosomes 1, 4, 6, 8, 11, and 12; notably, *qPN1.1* and *qPN8* were detected consistently in both years. By combining haplotype and expression analyses, *OsCKX11* was identified as the candidate gene for *qPN8*, while *LOC_Os01g07870* and *LOC_Os01g07950* were identified as candidate genes for *qPN1.1*. Significant differences were observed among the haplotypes of these candidate genes. Additionally, qRT-PCR results showed that *LOC_Os01g07870* expression levels were significantly lower in accessions with high panicle numbers compared to those with low panicle numbers.

**Discussion:**

To understand the natural biological function of these candidate genes, further research involving overexpression or silencing in rice is needed. Despite these challenges, our results will lay the foundation for further study of panicle development and provide valuable genetic resources for developing high-panicle-number rice cultivars using genetic engineering and molecular breeding.

## Introduction

Rice (Oryza sativa L.) is one of the major food crops in the world, sustaining more than half of the global population (Tilman et al., 2011). As the world’s population continues to increase, the area of arable land is gradually decreasing. Thus, providing higher rice yields is a top priority in the context of meeting the food demands of an increasing population (Seck et al., 2012; Liu et al., 2024). Rice yield is a complex agronomic trait composed of three components: panicle number, grain number, and grain weight (Xing and Zhang, 2010; Li P. et al., 2022). Among these factors, panicle number (PN) significantly contributes to rice yield, and maintaining stable tiller and PN is a key characteristic of ideal plant architecture (Jiao et al., 2010; Wang et al., 2018). Therefore, understanding the molecular mechanisms underlying panicle number is crucial for breeders aiming to improve rice yield.

The rice panicle is a compound raceme, composed of primary branches, secondary branches, and spikes on the branches. The developmental process of the rice panicle is highly complex. At the onset of the reproductive stage, the shoot apical meristem of rice transforms into inflorescence meristem, which produces primary branch meristems. Secondary branch meristems are sequentially produced on the primary branches, further differentiating into spikelet meristems and lateral spikelet meristems. Meanwhile, the apex of the primary branch meristem differentiates into a terminal spikelet meristem. These branches and the differentiated spikelet meristems ultimately form the basic structure of the rice panicle and determine the number of grains per panicle (Li et al., 2021). The panicle number is determined by the tiller number and how many of them can transition into effective tillers. The quantity of effective tillers directly influences the number of spikes per plant, subsequently affecting rice yield. Generally, the tiller (shoot branch) number keeps increasing during the vegetative growth stage after the initiation of the first tiller, and the older tiller will grow gradually along with the initiation of new tillers, and after a certain time of development, the oldest tiller will go into the reproductive development stage producing panicle (Wang et al., 2018). It was recently found that the dynamic change in tiller number (DCTN) pattern rather than maximum tiller number or effective tiller ratio is the determinant factor of high PN (Ma et al., 2020). The DCTN pattern that affords more panicles exhibits a period of stable tillering peak between 30 and 45 days after transplant, which was believed as an ideal pattern contributing to the steady transition from tiller development to panicle development (Ma et al., 2020). A recently identified quantitative trait locus (QTL) called *MORE PANICLES 3* (*MP3*) has been cloned as a single gene and shown to promote tillering and to moderately increase panicle number (Takai et al., 2023). This gene is an ortholog of the maize domestication gene *TB1*, and it has the potential to increase grain yield under ongoing climate change and in nutrient-poor environments (Takai et al., 2023).

Over the years, scientists have taken lots of efforts to uncover the genetic basis of tiller development in rice, and have cloned a large number of genes. For example, *MONOCULM1* (*MOC1*) and *MONOCULM3* (*MOC3*)/*TILLERS ABSENT1* (*TAB1*) are key factors in the initiation of tiller bud formation, and they are important for regulating tiller elongation. *MOC1* is specifically expressed in the leaf axillary meristem, and mutants of this gene cannot form tillers normally so the plants have only one main stem (Li et al., 2003). The *tab1-1* mutant is also unable to form tillers because it cannot form leaf axils (Tanaka et al., 2015). *MOC1* and *TB1*/*MOC3* also jointly upregulate *FLORAL ORGAN NUMBER1* (*FON1*) to participate in rice tiller elongation. Loss-of-function mutants of *FON1* form tiller buds normally, but the tillers are unable to elongate outward, thus resulting in fewer tillers. *MOC3* directly binds to the *FON1* promoter to enhance its expression and induce tiller elongation, while *MOC1* acts as a coactivator to enhance the transcriptional activation of *FON1* in the presence of *MOC3* (Shao et al., 2019). Moreover, various plant hormones, including auxin, strigolactone (SL), brassinosteroid (BR), gibberellin acid (GA), and cytokinin (CK), can regulate the growth and development of branches. For example, SLENDER RICE 1 (*SLR1*), a DELLA protein that acts as a repressor of GA signaling, can inhibit stem elongation while increasing the tiller number by supporting *MOC1* (Liao et al., 2019). Rice *CYTOKININ OXIDASE/DEHYDROGENASE 9 (OsCKX9)* acts upstream of the A-type response regulator *OsRR5* to inhibit CK synthesis (Duan et al., 2019). SL can induce the expression of *OsCKX9* to downregulate CK content, which in turn triggers the response of downstream genes and inhibits the growth of rice tillers (Duan et al., 2019). *OsAUX1* encodes an auxin influx transporter, and *SPL7* can directly binds to the *OsAUX1* promoter and regulates tillering in rice by altering *OsTB1* expression to modulate auxin signaling (Jia et al., 2024).

With the development of high-throughput sequencing and other biotechnological tools, the GWAS has been verified to be a useful approach for identifying genes, alleles, or haplotypes related to any traits of interest under complex environments. For example, Liu performed a GWAS of the tiller response to nitrogen that is most closely correlated with nitrogen-use efficiency in rice and identified a candidate gene *OsTCP19* (Liu et al., 2021). *Tiller Number 1* (*TN1*) was identified through genome-wide association study of tiller number (Zhang et al., 2023). Natural variation in *TN1* affects its interaction with TN1 interaction factor 1 (TIF1) to affect *DWARF14* expression and negatively regulate tiller number in rice (Zhang et al., 2023).

As outlined above, many genes involved in tiller development, including *MOC1*, *MOC3*, and *SLR1*, have been identified through mutant studies. However, due to their unfavorable phenotypes, such mutants have little value in crop improvement. Therefore, favorable alleles/haplotypes affecting tiller/panicle development must be mined from natural populations. In this study, we investigated the variation of panicle number using 411 *japonica* rice varieties, and performed the GWAS analysis to identify QTLs associated with panicle number. A total of seven QTLs were detected in two experimental years, and they were distributed on chromosomes 1, 4, 6, 8, 11, and 12, respectively. *qPN1.1* and *qPN8* were detected in both years, and we performed candidate gene analysis on these two important QTLs. Based on the results of haplotype and gene expression analysis, we predicted *OsCKX11*, which encodes a cytokinin oxidase/dehydrogenases, as the candidate gene for *qPN8*; *LOC_Os01g07870* and *LOC_Os01g07950* as candidate genes for *qPN1.1.* These findings will contribute to the elucidation of the genetic mechanism underlying panicle number and serve as a solid foundation for the development of high-panicle-number rice varieties.

## Methods

### Plant material

The natural population comprised 411 *japonica* rice varieties, which were collected from large collections of rice accessions preserved at the China National Rice Research Institute in Hangzhou, China. The fieldwork was conducted at the experiment station in Sanya (N18.25°, E109.51°) in 2021 and 2022. After germination, the rice seeds of each accession were transferred to the rice seedling bed for cultivation. The seedlings were transferred to the experimental field at 25 days old. The accessions were each planted in two replications, and a randomized plot experiment was conducted. Each replication contained 36 rice plants planted in six rows (6 × 6 matrix), using within-row and between-row distances of 20 cm. The panicle number survey was conducted at maturity stage. To avoid edge effects, where the growth of peripheral plants may be affected, we excluded the outer-row plants. Instead, we randomly selected 5 plants from the inner 4 × 4 area (16 plants in total) and used their average values for further analysis.

### Variant identification

We adopted the workflow by DePristo et al. (2011) for read mapping, variant discovery, genotyping and variant quality recalibration using the rice *Nipponbare* genome as the reference. We obtained SNP data for each rice variety using the default filter settings, retaining high-quality variants (Quality in Depth (QD) ≥ 20.0, ReadPosRankSum ≥ −8.0, Fisher’s (FS) ≤ 10.0, and Quality of Variant Loci (QUAL) ≥ the mean QUAL of all samples) and removing low-quality variants (QD < 20.0, ReadPosRankSum < −8.0, FS > 10.0, and QUAL < the mean QUAL of all samples). After merging the high-quality resequencing data of 407 accessions, we filtered all variants with the following parameters: minor allele frequency (MAF) ≥ 5%, heterozygous rate of per site ≤20%, and missing data rate <20%.

### Population structure, phylogenetic tree, kinship and linkage disequilibrium (LD) decay analysis

Population structure was investigated using ADMIXTURE (v.1.3.0) (Alexander et al., 2009) and the k (number of groups) was set from 2 to 10. The construction of phylogenetic trees was performed using FastTree based on the maximum likelihood method (Price et al., 2009). The exported phylogenetic tree was optimized by iTOL (https://itol.embl.de/itol.cgi). To assess the patterns of linkage disequilibrium (LD) decay in this *japonica* population, we computed the mean squared correlation coefficient (r^2^) values pairwise within 2 Mb using the software POPLDDECAY (v.3.40) (Zhang et al., 2019). Kinship analysis was conducted in software GAPIT (Wang and Zhang, 2021).

### Genome-wide association study

The Genomic Association and Prediction Integrated Tool (GAPIT3) was used to conduct the GWAS based on the compressed mixed linear model (MLM) program (Wang and Zhang, 2021). The Manhattan plot and Q–Q plot were generated with the qqman package of R (Stephen, 2014). The *p*-value <10^–4^ was chosen as the threshold to define significant SNPs.

### Candidate gene analysis

According to the GWAS results, we calculated the LD decay to discover the candidate regions of the significant loci using the LDheatmap software (Shin et al., 2006). And we conducted haplotype analysis of candidate genes using Beagle and DnaSP6 software (Browning and Browning, 2016; Rozas et al., 2017). The expression pattern of the genes in the candidate region was obtained from the RNA-seq database in the Rice Genome Annotation Project (http://rice.plantbiology.msu.edu/).

### RNA isolation and qRT-PCR

Total RNA was extracted using TRIzol reagent (Invitrogen Life Technologies, Shanghai, China) according to the manufacturer’s protocols. cDNA was synthesized using an RNA reverse transcription kit (Invitrogen Life Technologies, Shanghai, China). Quantitative real-time-polymerase chain reaction (qRT-PCR) was performed on an ABI 7500 Real-time PCR system (Bio-Systems:Life Technologies Holdings Pte. Ltd.). *OsActin1* (*LOC_Os03g50885*) was used as an internal reference. Relative transcript levels were calculated using the formula 2^–△△Ct^ (△△Ct = △Ct [test gene] –△Ct [reference gene]). Sequences of primers used are listed in Supplementary Table S4.

## Results

### Variation in panicle number in rice

**FIGURE 1:**
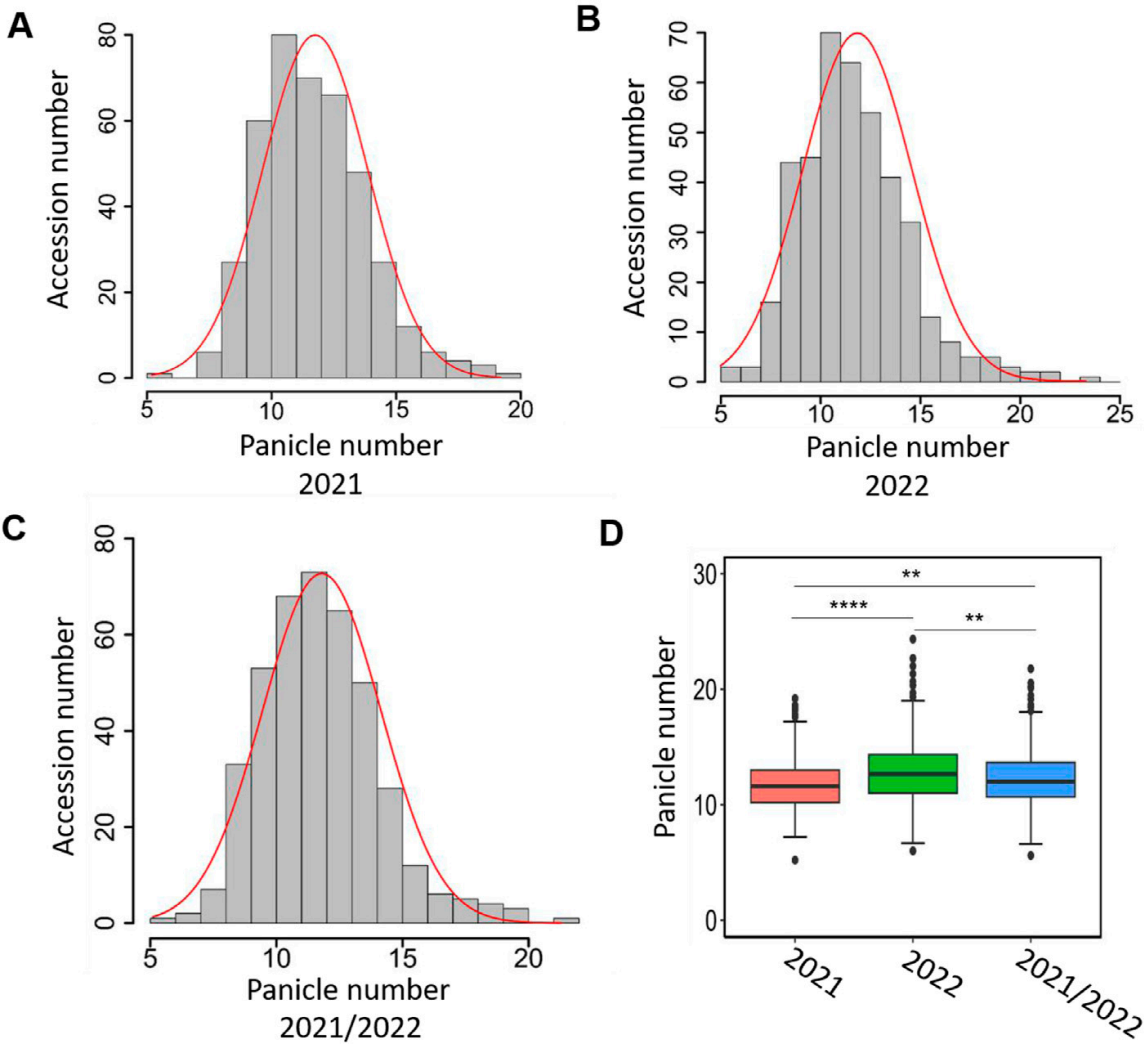
Variation of panicle number in *japonica* population. The distribution of panicle number in *japonica* population in 2021 **(A)**, 2022 **(B)**, and 2021/2022 **(C)**. The *X*-axis represents panicle number and *Y*-axis represents sample number. **(D)** Box plot of the phenotypic variation of panicle number. 2021/2022 represents the phenotypic values of 2021 and 2022 averaged. The asterisks *indica*te multiple significant difference detected by one-way ANOVA (**; *p* < 0.005; ****, *p* < 0.0001).

We surveyed panicle number over 2 years (2021 and 2022) for 411 *japonica* rice accessions. The panicle number showed significant variation among diverse rice accessions in 2021 and 2022 (Figures 1A, B; Supplementary Table S1). In 2021, the panicle number ranged from 5.2 to 19.2, with an average of 11.75 and a median of 11.6 (Figure 1A). The panicle number data in 2022 ranged from 6.0 to 24.3, the mean and median panicle number being 12.85 and 12.7 (Figure 1B). The panicle number among 411 lines for 2 years were positively correlated with each other, and the coefficients were high (r = 0.88, *p* < 0.001). In addition, we averaged the phenotypic values for 2021 and 2022 for each rice variety and displayed them as 2021/2022. In 2021/2022, the panicle number ranged from 5.6 to 21.77, with an average of 12.3 and a median of 12 (Figure 1C). The phenotypic data were consistent with normal distribution (Figures 1A–C). The panicle number showed statistical significance in 2021, 2022, and 2021/2022, especially in 2022 where the panicle number was significantly higher than in 2021 (Figure 1D).

### Polymorphic SNPs and genetic basis analyses of 411 *japonica* rice accessions

Whole-genome sequencing was performed on 411 *japonica* rice accessions. The average sequencing depth of the population was approximately 20.15×, the average genome coverage was 92.22%, and the average mapping rate was 96.13%, and the average error rate was 0.50%. After rigorous filtering and quality control of the genomic data of 411 accessions, a total of 743,678 high-quality SNPs were detected.

**FIGURE 2:**
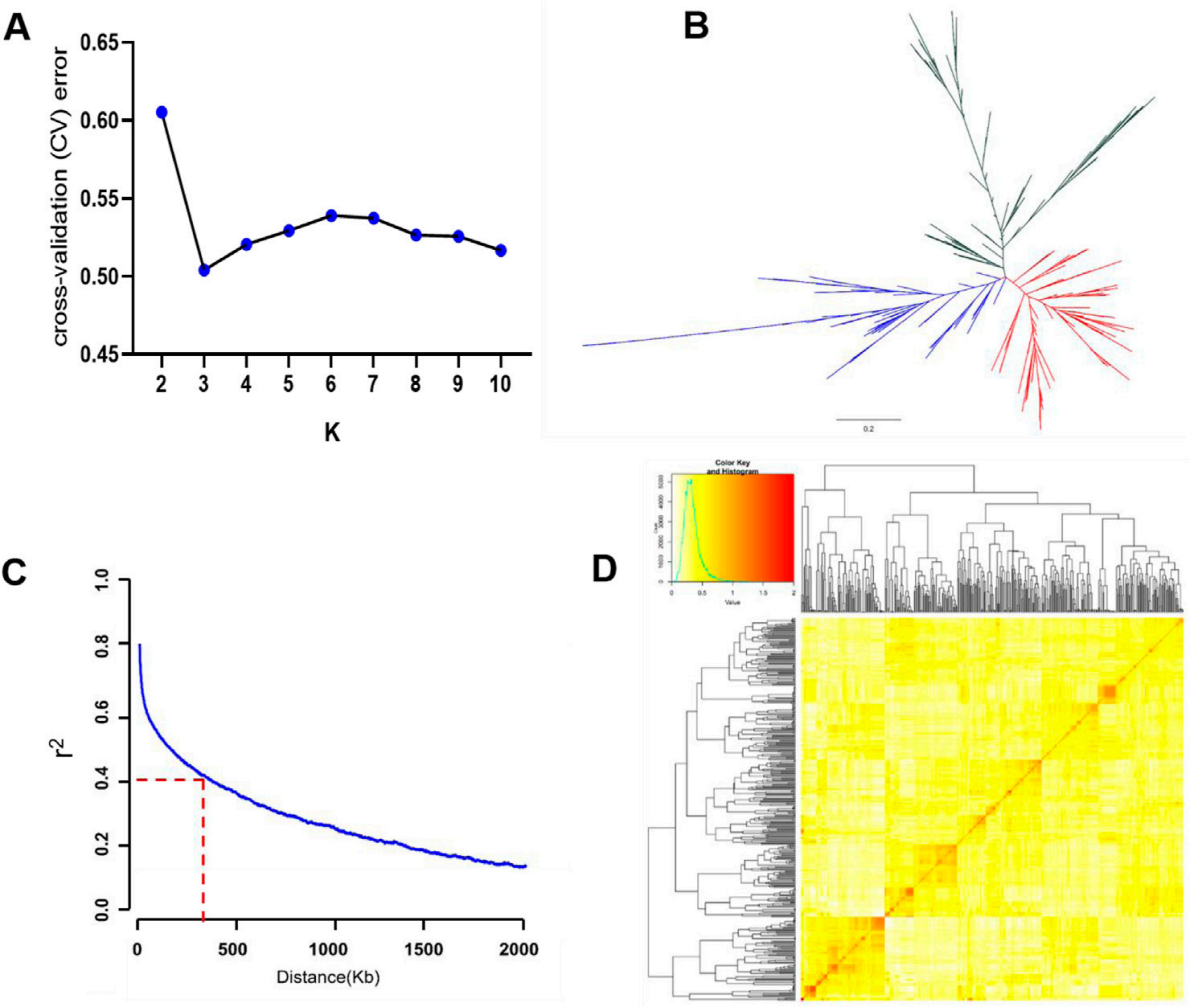
Genetic basis analyses of the 411 *japonica* varieties. **(A)** Distribution of cross-validation (CV) error values when the *japonica* varieties were divided into 2–10 subgroups. **(B)** Phylogenetic tree of the *japonica* varieties. **(C)** Decay of linkage disequilibrium (LD) in the whole genome of the *japonica* varieties. **(D)** Pairwise relative kinship analysis and the distribution of kinship coefficients of the *japonica* varieties.

We conducted population structure analysis with k from 2 to 10 using ADMIXTURE. The ideal K value with the least cross-validation error detected by the population structure analysis was determined as 3 (Figure 2A), so that the whole panel could be clustered into three main groups. The phylogenetic tree analysis divided the population into three groups (Figure 2B), consistent with the results of the population structure. The LD decay rate was measured as the chromosomal distance at which the average pairwise correlation coefficient dropped to half the value of the maximum r^2^. The result showed that the genome-wide LD decay rate was approximately estimated at 300 Kb where the r^2^ dropped to 0.4 (Figure 2C). On the basis of the genotype of the total 411 varieties, the pairwise relative kinship value was analyzed. 89.44% of the values ranged from 0 to 0.5, and 9.91% of the values ranged from 0.5 to 1 (Figure 2D). Moreover, only 0.65% of the values were larger than 1 (Figure 2D). The result suggested that there was weak relatedness among the *japonica* population, which is beneficial for subsequent GWAS analysis.

### Identification of the QTLs related to panicle number in rice

In order to investigate the genetic basis of panicle number in rice, we conducted a GWAS analysis to identify the SNP loci associated with panicle number in this natural population of 411 rice lines. A mixed linear model (MLM) was employed, incorporating the kinship matrix and PC matrix as covariates, thus reducing the interference of population structure in the GWAS analysis results.

**FIGURE 3:**
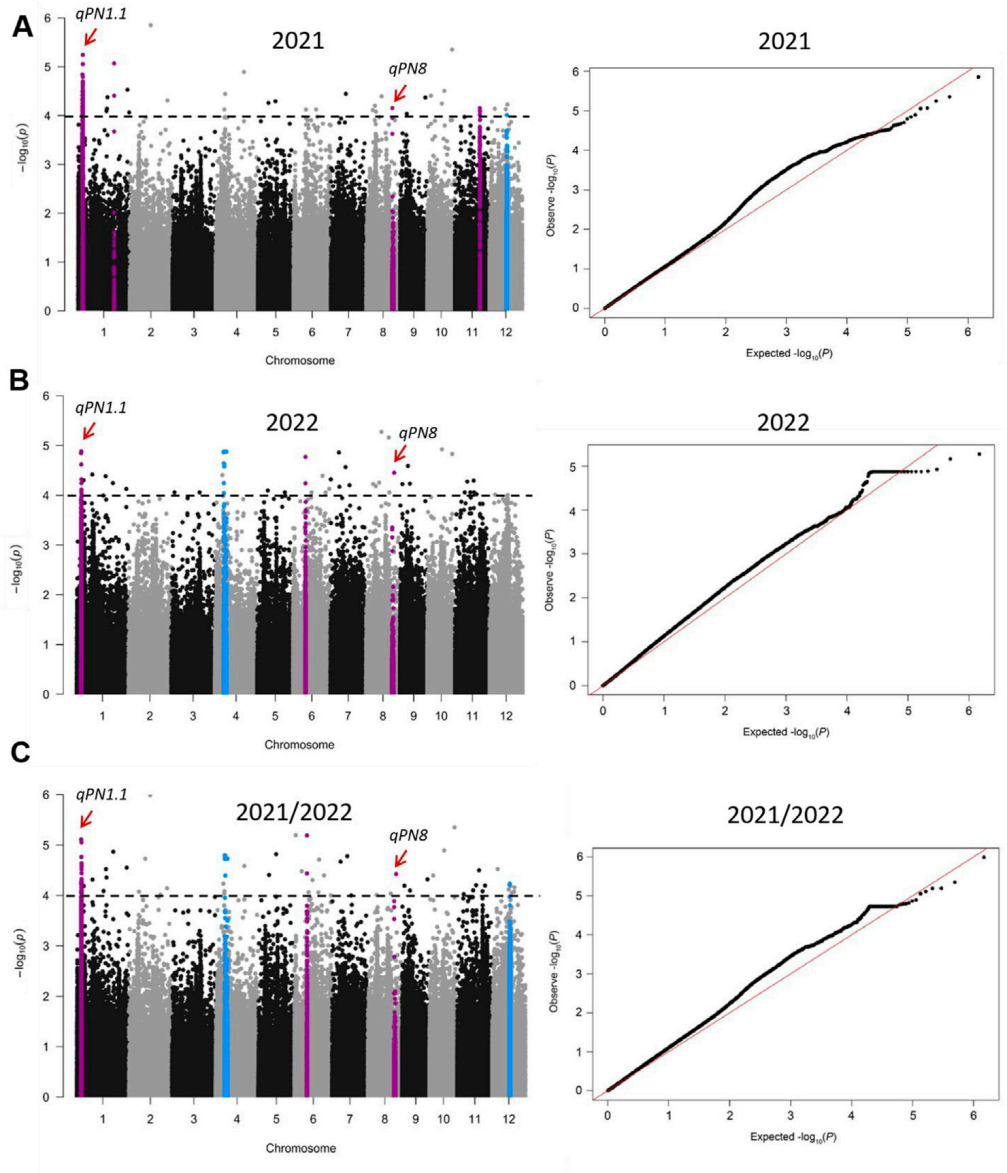
Genome-wide association study (GWAS) of panicle number using the mixed linear model (MLM). Manhattan plots and quantile–quantile (Q-Q) plot of GWAS analysis for panicle number in 2021 **(A)**, 2022 **(B)**, and 2021/2022 **(C)**. The horizontal black dashed lines showed the threshold at *P* = 0.0001. Purple and blue colors mark the quantitative trait loci (QTLs), where the blue QTLs overlap with previously reported panicle-number QTLs. The positions pointed by the red arrows are *qPN1.1* and *qPN8*. *PN* is the abbreviation of Panicle Number.

A total of 171, 88, and 157 SNPs were identified as having significant associations with panicle number in the year 2021, 2022, and 2021/2022 (Figures 3A–C; Supplementary Table S2). In line with the approach of a previous study (Lv et al., 2016), a region was considered as one QTL when more than two significant SNPs (*p* < 0.0001) were identified within a 200 Kb window. Based on this criterion, five QTLs were detected in 2021, namely, *qPN1.1, qPN1*.*2, qPN8, qPN11* and *qPN12*; four QTLs were detected in 2022, namely, *qPN1.1, qPN4, qPN6* and *qPN8*; and five QTLs were detected in 2021/2022, namely, *qPN1.1, qPN4, qPN6*, *qPN8 and qPN12* (Figures 3A–C; Table 1). A total of seven QTLs (*qPN1.1, qPN1*.*2, qPN4, qPN6*, *qPN8, qPN11, and qPN12*) were detected in this analysis (Figures 3A–C; Table 1), which were distributed on chromosomes 1, 4, 6, 8, 11, and 12. We compared the location of the QTLs detected in the current study with previously reported growth-related QTLs by the traditional method of Map-based cloning (http://www.gramene.org), and found two overlapped QTLs (Figures 3A–C; Table 1). The *qpn4.9* associated with panicle number was identified using a subset of 154 doubled haploid lines derived from a cross between two rice cultivars, CT9993 and IR62266 (Lanceras et al., 2004). This region spanned from 6574396 to 13635012 bp on chromosome 4 (Lanceras et al., 2004), overlapping with *qPN4* (Table 1). Another reported QTL *qPN12* associated with panicle number was detected from an interspecific BC_2_F_2_ population (Milyang 23/Oryza rufipogon), and its region spanned from 8826555 to 14450887 bp on chromosome 12 (Cho et al., 2003; Jin et al., 2011), overlapping with *qPN12* (Table 1). The other five QTLs, *qPN1.1, qPN1*.*2, qPN6, qPN8,* and *qPN11,* were newly found in this study. In addition, *qPN1.1* and *qPN8* were detected simultaneously in 2021, 2022, and 2021/2022 (Figures 3A–C; Table 1), implying that these QTL may have a significant effect on panicle number in rice.

**TABLE 1:** List of QTLs related to panicle number detected by GWAS analysis.

QTLs	Chr	QTL region (bp)	Lead SNP	*P*-value	Reported gene/QTL	Reported gene/QTL region (bp)
2021						
*qPN1.1*	1	3481185_4243959	1_3985818	5.67746E-06		
*qPN1.2*	1	30126040_30526212	1_30326040	8.49689E-06		
*qPN8*	8	21797950_23198042	8_21997950	6.99537E-05	*OsCKX11*	22616784_22619732
*qPN11*	11	20706068_21208693	11_20915884	7.05536E-05		
*qPN12*	12	13476849_14793388	12_14593388	5.71066E-05	*qPN-12*	8826555_14450887
2022						
*qPN1.1*	1	3481185_4222632	1_3905918	1.29812E-05		
*qPN4*	4	7334105_9761390	4_7538796	3.14277E-05	*qpn4.9*	6574396_13635012
*qPN6*	6	10189872_10590245	6_10389872	1.69048E-05		
*qPN8*	8	21797950_23817234	8_23617237	3.51111E-05	*OsCKX11*	22616784_22619732
2021/2022						
*qPN1.1*	1	3476704_4243959	1_3681185	7.71111E-06		
*qPN4*	4	7033052_9761390	4_7538796	1.85711E-05	*qpn4.9*	6574396_13635012
*qPN6*	6	10189872_10590245	6_10389872	6.41375E-05		
*qPN8*	8	21797950_23198042	8_23617237	3.74857E-05	*OsCKX11*	22616784_22619732
*qPN12*	12	14236827_14793388	12_14593388	5.85377E-05	*qPN-12*	8826555_14450887

### *OsCKX11*, the candidate gene of *qPN8*

**FIGURE 4:**
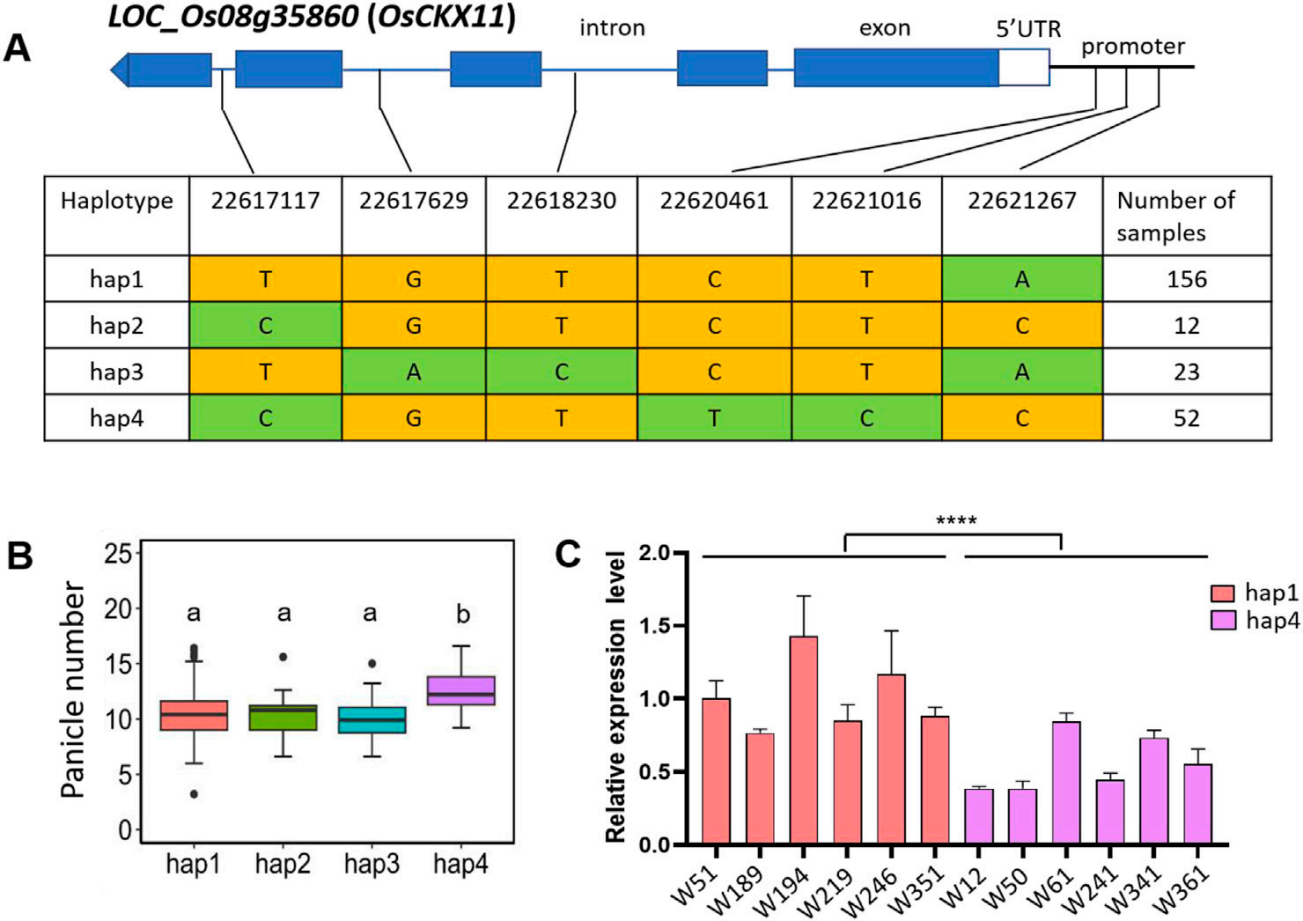
Haplotype and expression analysis of *OsCKX11* in *qPN8.* **(A)** Four haplotypes of *OsCKX11* based on 6 SNPs observed in all assessed rice varieties. Haplotypes with fewer than 5 varieties are not shown. Major and minor alleles are *indica*ted in yellow and green, respectively. **(B)** Box plots of panicle number based on the four haplotypes for *OsCKX11*. The letters on the bars *indica*te multiple significant difference detected by one-way ANOVA. **(C)** Expression levels of *OsCKX11* among germplasm materials; RNA was extracted from the bases of axillary buds of rice at 60 days after transplanting. Data are presented as means ± standard deviation (n = 3), and the *p*-value was determined by Student’s t-test (****; *p* < 0.0001).

The QTL *qPN8* was simultaneously detected in 2021, 2022, and 2021/2022, containing the previously reported gene *OsCKX11* (*LOC_Os08g35860*). *OsCKX11* encodes a cytokinin oxidase/dehydrogenases (CKX) and plays a role in delaying leaf senescence, increasing grain number, and coordinately regulating source and sink (Zhang et al., 2021). *Osckx11* presented with significantly increased branch, tiller, and grain number compared with the WT (Zhang et al., 2021). SNPs in the genome and 1 kb of upstream promoter region of *OsCKX11* were analyzed across 411 varieties. *OsCKX11* contains a total of six SNPs, the promoter contains three SNPs, genome contains three SNPs (Figure 4A). However, all three SNPs in the genome are located in introns. *OsCKX11* exhibits four major haplotypes (Figure 4A). It is noteworthy that the panicle number of hap4 was significantly higher than the other three haplotypes (Figure 4B), *indica*ting that *OsCKX11* is associated with panicle number. To investigate the causative SNP variation in *OsCKX11* responsible for panicle number, we used qRT-PCR to examine the expression level of *OsCKX11* between hap1 and hap4 lines. The results showed that the relative expression level of *OsCKX11* in hap4 was significantly lower than that in hap1 (Figure 4C), *indica*ting that SNP variations in the promoter may contribute more to the panicle number phenotype. In summary, these results suggest that *OsCKX11* is a potential candidate gene for *qPN8*.

### Candidate genes analysis of *qPN1.1*

**FIGURE 5:**
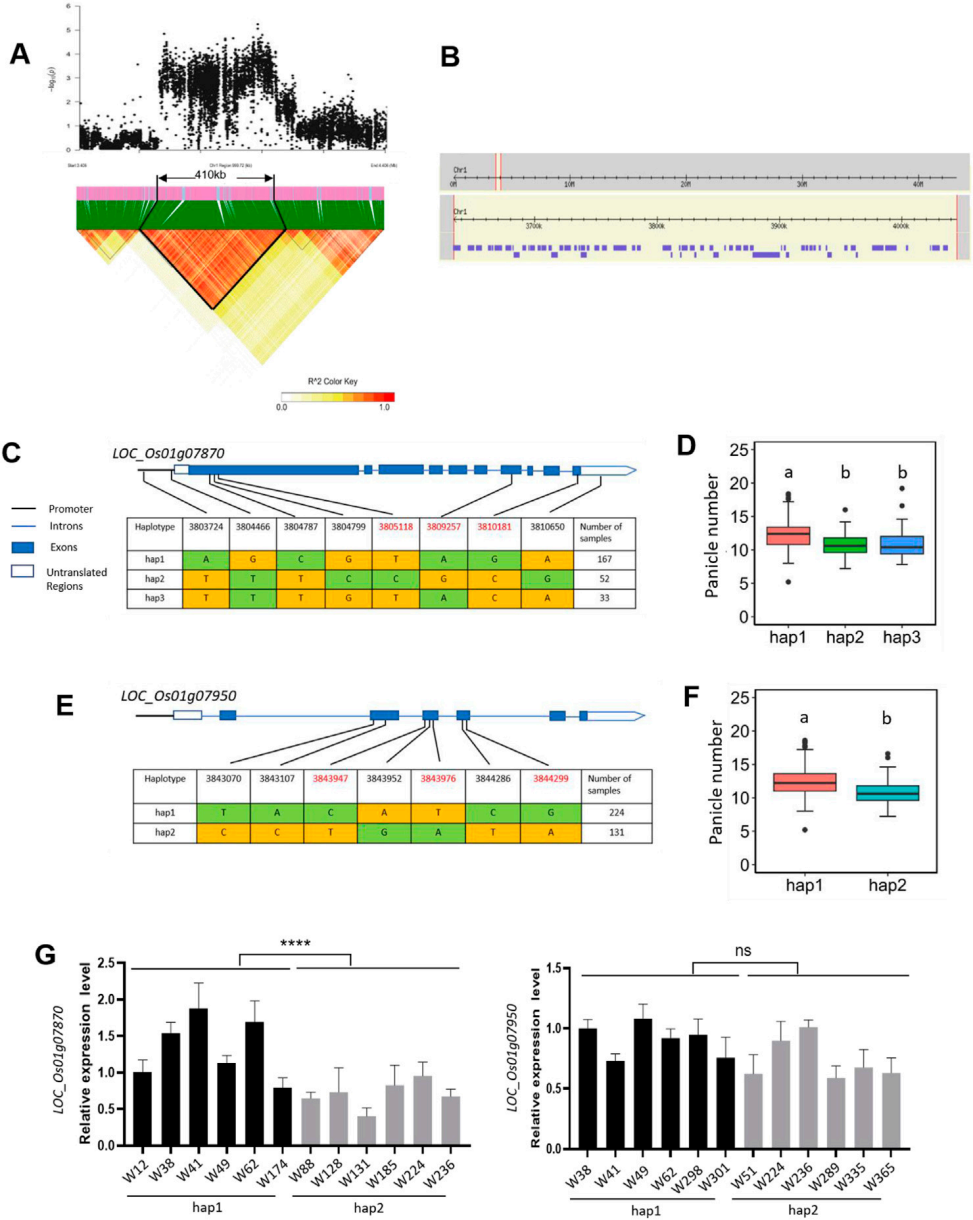
Identification of the candidate genes for the QTL *qPN1.1.* **(A)** Identification of the candidate region for *qPN1.1*. The pairwise LD was calculated with the r^2^ value (determination coefficient between the SNP states). **(B)** Identification of the genes in the candidate region of *qPN1.1*. Purple boxes represent genes. **(C)** Haplotypes of *LOC_Os01g07870.* Haplotypes with fewer than 5 varieties are not shown. Major and minor alleles are *indica*ted in yellow and green, respectively. SNPs in red font are non-synonymous SNPs. **(D)** Box plots of panicle number in accessions containing different *LOC_Os01g07870* haplotypes. The letters on the bars *indica*te multiple significant difference detected by one-way ANOVA. **(E)** Haplotypes of *LOC_Os01g07950*. Haplotypes with fewer than 5 varieties are not shown. Major and minor alleles are *indica*ted in yellow and green, respectively. SNPs in red font are non-synonymous SNPs. **(F)** Box plots of panicle number in accessions containing different *LOC_Os01g07950* haplotypes. The letters on the bars *indica*te multiple significant difference detected by one-way ANOVA. **(G)** Expression levels of candidate genes *LOC_Os01g07870* and *LOC_Os01g07950* among different germplasm materials. RNA was extracted from the bases of axillary buds of rice at 60 days after transplanting. Data are presented as means ± standard deviation (n = 3), and the *p*-values were determined by Student’s t-test (ns, *p* > 0.05; ****, *p* < 0.0001).

The QTL *qPN1.1* was simultaneously detected in 2021, 2022, and 2021/2022 and had the highest significance compared to the other QTLs. We estimated the candidate region of *qPN1.1* on chromosome 1 using the SNP data of 2021. Based on LD decay analysis, a total 410 kb region ranging from 3.63 to 4.04 Mb on chromosome 1 was identified as the candidate region which contained 1,165 SNPs, 63 genes (Figures 5A, B). Among the 1,165 SNPs, 465 are located within the promoter, UTR (Untranslated Region), or CDS regions of 39 genes (Supplementary Table S3). Generally speaking, genes that regulate panicle number should be expressed in the above-ground parts. Therefore, we screened the 39 genes using the RNA-seq data published by the Rice Genome Annotation Project and selected 12 genes with higher expression levels in above-ground parts for the candidate genes (Supplementary Table S3). From the SNP information, six of the 12 genes contained non-synonymous SNPs: *LOC_Os01g07740*, *LOC_Os01g07760*, *LOC_Os01g07870*, *LOC_Os01g07950*, *LOC_Os01g08130*, and *LOC_Os01g08200.* We performed haplotype analysis for the six candidate genes. No significant differences were found among the different haplotypes of *LOC_Os01g07740*, *LOC_Os01g07760*, *LOC_Os01g08130*, and *LOC_Os01g08200* (Supplementary Figure S1). Therefore, we consider *LOC_Os01g07870* and *LOC_Os01g07950* as candidate genes for *qPN1.1* and further to analyze them.

The full length of the *LOC_Os01g07870* locus is 4,518 bp, including ten exons and nine introns (Figure 5C). The *LOC_Os01g07870* gene encodes a 1506-amino acid ABC transporter protein with four conserved domains: two transmembrane domains and two ABC transporter-like domains (Supplementary Figure S2A). SNPs occurred in the upstream, exons, introns, and UTRs (Untranslated Regions) of the gene, resulting in the identification of three haplotypes (Figure 5C). The average panicle number of 167 accessions carrying hap1 was 12.25, while the corresponding averages values for carrying hap2 and hap3 were 10.89 and 11.01, respectively. There was no significant difference between hap2 and hap3. However, the average panicle numbers of hap2 and hap3 were significantly different from that of hap1 (Figure 5D). Additionally, we compared the expression levels of the gene in the two major haplotypes (hap1 and hap2) by qRT-PCR. *LOC_Os01g07870* showed significantly higher expression levels in high panicle number varieties (hap1) than in low panicle number varieties (hap2) (Figure 5G). These results suggest that increasing the expression of *LOC_Os01g07870* may increase panicle number. *LOC_Os01g07870* contained three non-synonymous SNPs. The SNP at 3805118 changed from base T to base C, resulting in a change from tryptophan (W) to arginine (R) at the amino acid 139. The SNP at 3809257 changed from base A to base G, resulting in a change from isoleucine (I) to valine (V) at the amino acid 1,313. The SNP at 3810181 changed from base G to base C, resulting in a change from arginine (R) to serine (S) at the amino acid 1,482. Amino acid 1,313 is located in the conserved ABC transporter-like domain 2, which is isoleucine (I) in rice but valine (V) in other species (Supplementary Figure S2B). Therefore, we speculate that the amino acid change from I to V at position 1,313 may not affect protein function.

For the gene *LOC_Os01g07950*, the genome length is 558 bp, including six exons and six introns. *LOC_Os01g07950* encodes a 186-amino acid glutaredoxin (*OsGRX1*) with a conserved glutaredoxin domain (Supplementary Figure S3). SNPs occurred only in the exons of the gene, resulting in the identification of two haplotypes (Figure 5E). The average panicle number of 224 accessions carrying hap1 was 12.31, while the corresponding average for those carrying hap2 was 10.90. The panicle number of hap1 lines was significantly higher than that of hap2 lines (Figure 5F). We also compared the expression levels of this gene in the two haplotypes but did not find significant differences (Figure 5G). LOC_Os01g07950 contained three non-synonymous SNPs. The SNP at 3,843,947 changed from base C to base T, resulting in a change from proline (P) to serine (S) at the amino acid 38. The SNP at 3,843,976 changed from base T to base A, resulting in a change from serine (S) to arginine (R) at the amino acid 47. The SNP at 3844299 changed from base G to base A, resulting in a change from valine (V) to isoleucine (I) at the amino acid 109. Valine (V) at position 109 is located in the conserved glutaredoxin domain, close to the active site CGFS, and is conserved in multiple species (Supplementary Figure S3). Therefore, we speculate that the mutation at amino acid position 109 is likely to affect the protein function of glutaredoxin.

## Discussion

Based on geographic distribution and genetic variation, Asian rice is classified into two subspecies: *indica* and *japonica* (Jing et al., 2023). Typically, *indica* rice exhibits stronger tillering ability, producing more tillers and panicles, which leads to significantly higher yields compared to *japonica* rice (Ma et al., 2020). In contrast, *japonica* rice has lower chalkiness, lower amylose content, and higher gel consistency, making its grain quality superior to that of *indica* rice (Wang et al., 2019). Therefore, increasing the tiller and panicle numbers in *japonica* rice is crucial for breeding high-yield, high-quality *japonica* varieties. Previous studies have identified favorable alleles controlling tiller and panicle numbers mainly in *indica* rice, such as *OsAAP5* (Wang et al., 2019) and *TN1* (Zhang et al., 2023). However, in this study, we focused on 411 pure *japonica* rice varieties and conducted a statistical analysis of their panicle numbers over two consecutive years. The results revealed significant variation in panicle numbers among these *japonica* varieties (Figures 1A–D), with the highest reaching 24.3, *indica*ting that favorable alleles for controlling panicle number also exist within *japonica* rice. Additionally, we observed a strong correlation in panicle numbers for the 411 *japonica* varieties between 2021 and 2022, although significant differences were still present between the 2 years (Figure 1D). Rice requires certain external conditions to enter the reproductive growth stage, such as suitable light, field water and nutrition levels, etc., (Domagalska and Leyser, 2011; Wang et al., 2018). Different environmental conditions may have an impact on the development of rice spikes. The significantly higher panicle number in 2022 than in 2021 in this study may also due to the different external environment (Figure 1D).

GWAS is useful for genetic dissection of complex traits in rice and has been widely proven by many studies (Wang et al., 2021; Yoshida et al., 2023; Wei et al., 2024). Furthermore, many novel genes have been identified using GWAS (Yano et al., 2016; Li J. et al., 2022; Yang et al., 2024). Here, we conducted a genome-wide association study (GWAS) on the number of panicles in 411 *japonica* rice accessions and detected a large number of significant SNPs, especially in 2021, where 171 significant SNPs were identified (Figures 3A–C). The detection of a high number of significant SNPs in GWAS is not generally considered as an ideal result, however, for complex traits such as yield, plant height, tiller number, and panicle number, which are controlled by multiple genes, it is common to associate a large number of SNPs during association analysis (Huang et al., 2010; Yano et al., 2016; Ma et al., 2020; Zhang et al., 2023). Additionally, population structure is another important factor that influences GWAS results, as it increases the rate of false positives. Population structure analysis revealed that the 411 *japonica* rice accessions used in this study could be divided into three subgroups (Figure 2A), and the presence of this structure may have also affected the GWAS results.

To eliminate the potential impact of different growth conditions on GWAS results, some studies integrate phenotypic data from varying conditions to calculate averages for more accurate GWAS results (Ye et al., 2022; Wei et al., 2024). Therefore, we used the average of panicle number from 2021 to 2022 as an additional dataset (2021/2022) for GWAS analysis. A total of seven QTLs were detected using three sets of panicle number data (2021, 2022, and 2021/2022) (Figures 3A–C; Table 1). In all identified QTLs, *qPN1.1* and *qPN8* were detected simultaneously at 2021, 2022, and 2021/2022, considered to be the main effect QTLs of panicle number that less effected by environmental factors. Other QTLs were detected in only 1 year, and it is hypothesized that the effects of these QTLs may be relatively weak or more easily influenced by environmental factors. Moreover, two QTLs were co-located with previously reported panicle-number-related QTLs identified by traditional mapping methods (Table 1), *indica*ting that our GWAS results are quite reliable.

Cytokinins (CKs) play a crucial role in stem cell division, panicle development, plant architecture, nitrogen utilization, and environmental stress responses. CKX genes encode cytokinin oxidase/dehydrogenases, which regulate the levels of cytokinins in tissues and are vital for determining agronomic traits in crops. In rice, 11 CKX family proteins have been identified. The allele variation of *OsCKX2/Gn1a* promotes CK accumulation, increases the number of spikes and grains, and enhances lodging resistance (Tu et al., 2022). *OsCKX3*-mediated CK accumulation regulates leaf sheath development and negatively modulates leaf angle (Huang et al., 2023). *Osckx1 Osckx2* double mutant shows a reduction in tiller number, an increase in panicle size, a decrease in seed setting rate, and an increase in thousand-grain weight (Rong et al., 2022). In contrast, the *Osckx4 Osckx9* double mutant exhibits an increase in tiller number, smaller roots and panicles, and a decrease in both seed setting rate and thousand-grain weight (Rong et al., 2022). The *Osckx11* mutants exhibited enhanced sink strength by increasing the tiller and grain number, consequently leading to elevated yield (Zhang et al., 2021). This evidence suggested that *OsCKX11* has great potential in breeding for high crop yield. In this study, we identified a favorable allele of *OsCKX11* affecting panicle number in a natural population. The expression of the favorable allele was significantly lower and associated with larger panicle numbers (Figure 4C), similar to the *Osckx11* mutant (Zhang et al., 2021). The promoter of the favorable allele contains two unique SNP sites that are likely to be binding targets for certain transcription factors (Figure 4A). Mutations in the binding targets of transcription factors in natural populations can affect gene expression, as reported in several studies. For example, a single nucleotide variant in the promoter of the *bsr-d1* gene inhibits the binding of MYB transcription factors, resulting in reduced gene expression (Li et al., 2017). 272 bp insertion/deletion at 387 bp upstream of the start codon in the *Tn1a* promoter confers a differential transcriptional response and results in a change in tiller number (Yang et al., 2024). Although yield increased after *OsCKX11* knockdown, fertility and thousand-grain weight were reduced, which may be an adverse effect of complete deletion of *OsCKX11*. Our newly identified favorable allele is a weak allele that offers the possibility of correcting the problems of low fertility and seed size.

ABC proteins are involved in regulating diverse biological processes in plants, such as growth, development, uptake of nutrients, tolerance to biotic and abiotic stresses, shape, and size of grains etc (Dahuja et al., 2021). *Arabidopsis ATP-binding cassette* (*ABC*) *transporter subfamily G14* (*AtABCG14*) participates in transporting multiple cytokinins, and *atabcg14* mutants show retardation of growth and development (Zhao et al., 2023). ABCB transporters also play a very important role in the distribution of auxin inside the plant (Geisler et al., 2017). By integrating expression and haplotype analysis, we screened one strong panicle-number-related genes, *LOC_Os01g07870* (Figure 5). *LOC_Os01g07870* encodes an ABC transporter, thus we speculate that *LOC_Os01g07870* may affect panicle/tiller development by mediating the transport of plant hormones.

Another candidate gene, *LOC_Os01g07950* (*OsGRX1*) encodes a glutaredoxin (Rouhier et al., 2006). Glutaredoxins (Grxs) are a ubiquitous group of oxidoreductase enzymes that are important in plant growth and development (Wu et al., 2017). GRXs have long been recognized as crucial regulators of meristem development, since they control ROS status of the cells that, in turn, regulate auxin signaling and cell replicative cycles. The role of GRXs in plant development was first described in *Arabidopsis* upon identification of so-called *roxy1* mutants, which display reduced numbers of petal primordia and abnormalities during subsequent petal development (Xing et al., 2005). The close homolog of *ROXY* in maize, *MALE STERILE CONVERTED ANTHER1* (*MSCA1*), has been reported to play a crucial role in determining male germline fate and shoot apical meristem size (Kelliher and Walbot, 2012). Both auxin sensitivity and polar auxin transport are perturbed in the *grxs17* mutants of *Arabidopsis*, resulting in defects in cell proliferation and cell cycle control, particularly under high-temperature conditions (Cheng et al., 2011). In addition, *grxs17* mutants exhibit smaller shoot apical meristems, altered photoperiod, and delayed bolting (Knuesting et al., 2015). Therefore, we hypothesize that *LOC_Os01g07950* may also play a role in meristem maintenance and panicle development.

To understand the natural biological function of these candidate genes, further research involving overexpression or silencing in rice is needed. Despite these challenges, our results will lay the foundation for further study of panicle/tiller development and provide valuable genetic resources for developing high-panicle-number rice cultivars using genetic engineering and molecular breeding.

## Data Availability

The original contributions presented in the study are publicly available. This data can be found here: https://bigd.big.ac.cn/gvm/getProjectDetail?Project=GVM000882.
